# Hormonal priming with indole-3-acetic acid modulates antioxidant defense, gene expression, and physiological responses under salinity in *Limonium* spp.

**DOI:** 10.3389/fpls.2026.1765513

**Published:** 2026-04-10

**Authors:** Nishtha Vashishta, Megha Katoch, Chhering Youdon, Deepti, Bhavya Bhargava

**Affiliations:** 1 Floriculture Laboratory, Agrotechnology Division, Council of Scientific and Industrial Research (CSIR), Institute of Himalayan Bioresource Technology (IHBT), Palampur, H.P, India; 2 Academy of Scientific and Innovative Research (AcSIR), Ghaziabad, Uttar Pradesh, India

**Keywords:** gene expression, indole acetic acid, *Limonium* spp., salt stress mitigation, salt threshold level

## Abstract

**Introduction:**

Soil salinization is a major environmental challenge, limiting agricultural productivity and threatening ecological sustainability worldwide. *Limonium* spp., a halophytic genus with moderate salt tolerance, holds promise for revegetation and restoration of saline-affected lands. However, under severe salinity, its growth and physiological performance are adversely affected, highlighting the need for strategies to enhance its resilience. In this study, the potential of IAA priming to mitigate salinity-induced damage was evaluated in two *Limonium* cultivars, sky light and deep blue.

**Methods:**

One-month-old plants were exposed to 250 mM NaCl, with or without prior priming with IAA. To evaluate the effect of IAA on salt stress mitigation, morphological, physiological, biochemical and molecular investigations were carried out.

**Results:**

Salt stress markedly impaired plant growth, reduced relative water content, chlorophyll content, and photosynthetic efficiency, while increasing Na^+^ accumulation, electrolyte leakage, lipid peroxidation, and oxidative stress. By contrast, IAA priming (20mM) alleviated these detrimental effects by enhancing osmotic adjustment, maintaining K^+^/Na^+^ homeostasis, improving chlorophyll retention, and reinforcing the antioxidant defense system. Proline accumulation, total soluble protein content, and stomatal regulation were also positively influenced by IAA treatment. Also, the expression of salt-responsive genes (RD22, RD29A, SOS1, AtP5CS1, LbMYB48, and LbAPX3) was upregulated under salinity, with a stronger induction observed in IAA-primed plants.

**Discussion:**

These findings highlight the regulatory role of IAA in modulating physiological, biochemical, and molecular responses to salinity stress, thereby enhancing the salt tolerance of limonium. The study underscores the potential of IAA priming as a practical strategy for improving the performance of plants under salinity to support the restoration of saline ecosystems.

## 1 Introduction

Abiotic stresses, particularly salinity, represent one of the most severe growing global issues. It is causing soil degradation, desertification, and loss of arable land, thereby threatening agricultural sustainability and global food security (Guo et al., 2017). Currently, about 930 million hectares area worldwide is affected by salinity (Zaman et al., 2018). Rapid urbanization, unsustainable irrigation, and climate change continue to worsen the problem, leading to a yearly loss of approximately 1% of arable land and an expansion of saline areas at a rate of 3 ha min^-1^ (Honfi et al., 2023). If current trends continue, salinity-affected land could reach over 16 million hectares by 2050 (Kumar et al., 2022). In plants, salt stress disrupts water uptake (osmotic stress), disturbs ion balance through excessive Na^+^ and Cl^-^ accumulation (ionic toxicity), and enhances the production of reactive oxygen species (oxidative stress). Thus, impairing germination, seedling vigor, photosynthesis, growth, and ultimately crop yield (Demidchik and Shabala, 2017).

Halophytic plants possess the extraordinary capacity to complete their life cycles in the extreme saline environments with extreme species surviving at concentrations up to 2000 mM NaCl (Alhaddad et al., 2021; Rahman et al., 2021). These species are indispensable for restoring high-salinity ecosystems and improving soil fertility by actively extracting salts from the soil surface. A primary example of such resilience is *Aeluropus lagopoides*, a salt-secreting perennial grass that thrives in up to 1M NaCl by maintaining ionic homeostasis and strategically reducing gas exchange to minimize Na^+^ uptake (Ahmed et al., 2013). Unlike glycophytes that suffer damage at 50 mmol NaCl, halophytes can grow, reproduce, and complete their life cycle at 400 mmol or even more (Ibraheem et al., 2025). To cope with such high salinity levels, halophytes such as *Suaeda maritima*, *Salicornia europaea*, and *Tetraena qatarensis*, have evolved various physiological, metabolic, and molecular adaptive strategies that protect them from the harsh saline environment (Pungin et al., 2023; Yasseen and Thani, 2022; Ghanem et al., 2021).

The genus *Limonium* (Plumbaginaceae) represents one of the most biodiverse and ecologically significant groups within the halophytic flora, inhabiting saline soils, salt marshes, and coastal habitats (González-Orenga et al., 2021; Gancedo et al., 2023). As recretohalophytes, approximately 370 *Limonium* species (Honfi et al., 2023) utilize specialized salt glands and bladders to actively excrete toxic ions from their leaf tissues. Among these, species such as *L. gmelinii* exhibit a broad tolerance spectrum (50–400 mM NaCl) (Mi et al., 2021), however, physiological functions in many *Limonium* species such as *Limonium sinuatum* are typically hindered beyond 200 mM NaCl as plants redirect metabolic energy from biomass accumulation toward the activation of defense mechanisms.

Beyond their role as pioneer species for saline land restoration, *Limonium* species are highly valued as landscape plants and cut flowers due to their vibrant inflorescences and long vase life. They also serve as reservoirs for secondary metabolites, including flavonoids, phenolics, and tannins, with significant pharmacological potential (Xiaoke et al., 2025). Despite their importance, the specific mechanisms underlying salt tolerance, stress signaling, and detoxification pathways in many halophytic species remain largely unclear. Consequently, identifying the threshold responses and signaling networks in species like *Limonium bicolor* and *Limonium sinuatum* is critical for advancing sustainable agriculture in salt-affected environments.

As typical recretohalophytes, *Limonium* species are mostly C_3_ perennial herbs or shrubs and have special structures such as salt bladders and salt glands for salt excretion, which glycophytes do not have. Salt bladders are modified, balloon-like trichomes that store salt on the leaf surface, while salt glands are stable multi-cellular units that actively remove toxic ions. In simple terms, bladders store salt, whereas glands continuously release it (Al Hassan et al., 2017). However, this excretory capacity must be synchronized with internal osmotic adjustments to prevent leaf cell dehydration. For this, *Limonium* uses two approaches: it stores inorganic ions (Na^+^, Cl^-^) in vacuoles for low-energy osmotic balance, and at same time produces energy demanding organic osmolytes (Shabala et al., 2014). In recent years, plant MYB transcription factors are a popular class of transcription factors related to the plant growth, metabolism, cell form and tolerance to abiotic stress (Wang et al., 2021). An R1-type MYB transcription factor gene was screened from *L. bicolor*, named LbMYB48. This gene act as a key switch that increases salt gland density and enhance sodium secretion by controlling epidermal development genes (such as LbCPC-like and LbDIS3) along with stress-response genes like LbSOSs, LbRLKs, and LbGSTs. In addition, LbMYB48 helps maintain osmotic balance and ion homeostasis, likely through the abscisic acid (ABA) signaling pathway. This coordination allows the plant to remove excess salt while keeping cells functional under high salinity conditions (Han et al., 2022).

According to Moreno et al., who classified various perennial *Limonium* species based on morphological traits and plant-soil relationships, *L. angustebracteatum* typically inhabits soils characterized by high electrical conductivity (EC), elevated Na^+^, K^+^, Cl^-^, concentrations, and a high exchangeable sodium percentage (ESP) (Moreno et al., 2018). Similarly, other species within the genus, including *L. virgatum*, *L. santapolense*, *L. girardianum*, and *L. narbonense*, have demonstrated a remarkable ability to thrive in saline environments (below 200 mM salinity threshold) (Orenga et al., 2019; Calone et al., 2023).

Plants mitigate salt stress through coordinated molecular, physiological, biochemical, and morphological defense mechanisms, including hormonal signaling, osmolyte accumulation, ion compartmentalization, and antioxidant enzyme activation (Demidchik and Shabala, 2017). Among phytohormones, indole-3-acetic acid (IAA) plays a pivotal role in regulating growth and adaptive responses under saline conditions (Jing et al., 2023). Acetic acid improves plant tolerance to abiotic stresses by adjusting key physiological and biochemical processes. These include root and shoot growth, photosynthesis, water-use efficiency, stomatal conductance, chlorophyll synthesis, leaf water status, nutrient-use efficiency, and the activation of antioxidant defense mechanisms (Rahman et al., 2019b; 2021; Utsumi et al., 2019; Hossain et al., 2020; Saberi et al., 2021). Supporting this, Zhang et al. (2021) showed that application of 20 mM acetic acid enhances salt tolerance in perennial ryegrass by inducing LpPDC1, maintaining a favorable K^+^/Na^+^ ratio, increasing SOD and CAT activities, and reprogramming hormonal balance, with higher auxin levels and lower ABA. Emerging evidence also suggests that IAA-related regulation may involve epigenetic mechanisms, such as histone acetylation, chromatin remodeling, and modulation of Ca²^+^/Mg²^+^ homeostasis, which helps plants adapt to stress over time (Park et al., 2021). Together, these finding highlight that IAA plays multiple role in improving salt tolerance and can be used to enhance stress resilience in crops such as *Arabidopsis thaliana*, *Triticum aestivum*, and *Vigna radiata* (Aizaz et al., 2023).

Given their ecological and ornamental importance, and their role as pioneer species in salt-affected ecosystems, improving salt tolerance in *Limonium* is important. Although the use of auxins is known to reduce salt stress in model plants and crops, how auxin work in ornamental halophytes is still not well understood. In this study, we examine the role of IAA priming in improving salt tolerance in two *Limonium* varieties, *L. bicolor* ‘sky light’ and *L. sinuatum* ‘deep blue’. We used growth and physiological traits, biochemical markers, microscopic observations, and expression of salt-responsive genes to understand how IAA supports stress adjustment. We hypothesize that IAA application improves salinity tolerance by regulating ion homeostasis, antioxidative capacity, and expression of key stress-regulatory genes. This study helps clarify how hormones control stress responses in halophytes and provides useful insights for developing salt-tolerant plant for saline agriculture and landscape restoration.

## 2 Material and methods

### 2.1 Plant materials and growth conditions

Three varieties of *Limonium*, namely ‘sky light’, ‘silvery pink’ (*Limonium bicolor*), and ‘deep blue’ (*Limonium sinuatum*), were used in this study. Twenty-days-old seedlings were transplanted into plastic pots (16 cm diameter × 12 cm height) filled with a mixture of field soil and farmyard manure (FYM). Plants were grown under greenhouse conditions at CSIR-IHBT (Day temperature: 25 ± 2^0^C; Night temperature: 12 ± 2^0^C). Standard agronomic practices were followed.

### 2.2 Determination of salt threshold level

To assess salt tolerance thresholds, three-week-old seedlings at the 10–12 leaf stage were treated with different salt concentration i.e. 50, 100, and 250 mM NaCl every alternate day for four weeks. Control plants received only distilled deionized water. Dry weight of leaves and roots were measured following the method of Huang et al. (2019). For each variety, five biological replicates were analyzed for each treatment, and the mean values were calculated for each variety. The salt tolerance threshold was determined by plotting a regression curve correlating NaCl concentrations with dry weight (DW), and identifying the concentration at which plants retained 50% of their DW compared to the control. The Salt Tolerance Index (STI) was calculated as:


$$STI\;\left(\%\right)=\frac{DW\left(salt\;stress\right)}{DW\left(control\right)}\;\times 100$$

### 2.3 Experimental design and treatments

Based on preliminary results, 250 mM NaCl, causing ~50% growth reduction, was selected for subsequent experiments. Among the three varieties, ‘sky light’ and ‘deep blue’ showed higher tolerance and were selected for detailed analysis. The method for acetic acid treatment was selected according to a study reported by Kim et al. (2017).

For the priming treatment, each pot received 200 mL of acetic acid solution (10 or 20 mM) once a day for three days. Any excess solution was allowed to drain out by capillary action using a paper towel placed at the bottom of the pots. The plants were then maintained in the polyhouse for another five days before salt stress was imposed. To induce salinity stress, plants were irrigated with 200 mL of 250 mM NaCl per pot every alternate day for 15 days, while control plants received the same volume of water. Throughout the experimental period, plants were grown under a 15-h light/9-h dark photoperiod. Soil salinity was checked weekly by measuring electrical conductivity (EC) following the US Salinity Laboratory method to ensure stable salt levels (Staff, 1954). The experimental design consisted of six treatments: (i) Control (T1), (ii) 10 mM IAA (T2), (iii) 20 mM IAA (T3), (iv) 10 mM IAA + 250 mM NaCl (T4), (v) 20 mM IAA + 250 mM NaCl (T5), and (vi) 250 mM NaCl (T6). After completion of the treatments, plants were sampled from each biological replicate, and all treatments were performed in three independent biological replications. The collected fresh tissues were immediately frozen at -80 °C and stored until further use for biochemical assays, morphological evaluations, and gene expression studies.

### 2.4 Growth parameters

The fresh weight of root and leaves were measured using an electronic balance immediately after the plants were uprooted. The roots were rinsed with distilled water before weighing. For dry weight, samples were oven-dried at 70 °C for 72 hours. Leaf succulence was calculated as fresh weight per unit leaf area (Silveira et al., 2009) using formula: Leaf Succulence (mg FW cm^-^²) = Leaf fresh weight/Leaf area. Nine leaves per treatment were analyzed (three leaves from each of three trifoliates).

### 2.5 Leaf relative water content

Leaf relative water content (RWC) was determined following the method described by Yildirim et al. (2009) using the formula.


$$RWC\;\left(\%\right)=\frac{FW-DW}{TW-DW}\times 100$$

where FW is fresh weight, TW is turgid weight after soaking fresh leaves in water for 10 hours, and DW is dry weight after oven-drying fresh leaves at 74 °C for 24 h.

### 2.6 Membrane stability index

MSI was assessed following Almeselmani et al. (2011). Leaf segments (0.1 g) were incubated in distilled water at 40 °C for 30 min (C1), and at 100 °C for 15 min (C2). Electrical conductivity was measured using a conductivity meter (PC 700, Eutech Instruments, Singapore). MSI was calculated as:


MSI (%)=1−C1C2× 100

### 2.7 Mineral analysis

To assess ion contents, dried and fully expanded leaves and roots were ground, and a 0.5 g sample was placed in a test tube. The root and leaf samples were digested at 80 °C in a mixed acid solution consisting of nitric acid: perchloric acid in a 9:4 ratio (v/v) until the desired result was obtained (Allen et al., 1985). After cooling, the extract was filtered using Whatman No. 42 filter paper, and the filtrate was diluted to 20 ml with double-distilled water and make final volume of 100ml and the Na^+^ and K^+^ concentrations were measured using a flame photometer (Flame Photometer 410, Sherwood Scientific Ltd).

### 2.8 Gaseous exchange attributes

Net photosynthetic rate (P_n_), stomatal conductance (gs), and transpiration rate (E) were measured using a portable IRGA (Li-COR 6400, USA). Measurements were taken on fully expanded leaves between 09:00 am and 11:00 am under standardized conditions of 1200 µmol m^-^² s^-^¹ PPFD, 25 °C temperature, 65% RH, and 6 cm² leaf chamber area. Leaves were equilibrated for 90 seconds prior to measurement.

### 2.9 Biochemical analysis

#### 2.9.1 Chlorophyll and carotenoid content determination

Chlorophyll and carotenoid contents were estimated from homogenized fresh leaves using 80% (v/v) acetone extract. Absorbance was recorded spectrophotometrically at 663 nm, 645 nm, and 480 nm, respectively. The results were expressed as milligram (mg) per gram dry weight (mg g ^−1^ DW) (Maxwell and Johnson, 2000).

#### 2.9.2 DPPH radical scavenging capacity

The DPPH (2,2-diphenyl-1-picrylhydrazyl) radical scavenging activity was evaluated using method given by Thaipong et al., 2006. The DPPH scavenging activity was determined by calculating the percentage (%) inhibition of DPPH using the formula:


$$Percent\;Inhibition\;\left(\%\right)=\frac{Absorbance\;of\;Control-Absorbance\;of\;Sample}{Absorbance\;of\;Control}\times 100$$

#### 2.9.3 Proline content

The free proline content was quantified using a modified method by Bates et al. (1973). The proline concentration was determined using a calibration curve for proline as mg g^−1^ FW (fresh weight).

#### 2.9.4 Lipid peroxidation (MDA)

Lipid peroxidation was quantified by measuring malondialdehyde (MDA) levels using the thiobarbituric acid reactive substances (TBARS) assay (Hongbo et al., 2005).


$$MDA\;levels\;\left(nmol\;g^{-1}FW\right)\;were\;calculated\;=\frac{\left(A532-A600\right)N}{155W}$$

Here 155 is the absorption coefficient, N is the total volume of supernatant, W is the fresh weight of plant material (g).

#### 2.9.5 Total soluble protein

The soluble protein content was quantified utilizing the Bradford method (Bradford, 1976), employing bovine serum albumin as the standard reference.

#### 2.9.6 Antioxidant enzyme activity

The superoxide dismutase (SOD) activity was estimated using the protocol given by Giannopolitis and Ries (1997), based on the inhibition of photochemical reduction of nitro blue tetrazolium (NBT). The 200 µL reaction mixture contained methionine (197.8 mM), NBT (1.66 mM), riboflavin (175 µM), 50 mM phosphate buffer, and 100 µL enzyme extract. The mixture was exposed to fluorescent light (86.86 µmol m^-^² s^-^¹) for 10–20 min, and the reaction was monitored by a color change. Absorbance was measured at 560 nm, and one unit of SOD activity was defined as the amount of enzyme required to inhibit NBT reduction by 50%.

Catalase (CAT) activity was determined following the method of Cakmak and Marschner (1992). Fresh leaf tissue (100 mg) was homogenized in a hand mortar with 50 mM phosphate-potassium buffer (pH 7.8) containing 2 mM EDTA, 1 mM DTT, 1 mM PMSF, 5% Triton X-100, and 2% PVPP. The homogenate was centrifuged at 13,000 × g for 20 min at 4 °C, and the supernatant was collected for analysis. CAT activity was assayed in a 200 µL reaction mixture containing enzyme extract, 50 mM phosphate buffer (pH 7.0), and 150 mM H_2_O_2_. The reaction was initiated by adding the enzyme extract, and the decrease in absorbance at 240 nm was recorded over 5 min. One unit of CAT activity was defined as the amount of enzyme decomposing 1 mmol H_2_O_2_ per minute at 25 °C.

### 2.10 Scanning electron microscopy

Fresh leaves samples were taken and the epidermal layer from the lower surface was carefully peeled. The samples were then analyzed using scanning electron microscopy (HITACHI S-3400N). The total number of stomata in each visual field was counted manually.

### 2.11 Quantitative real time-PCR validation of salt stress tolerant genes

Total RNA was isolated from young leaves using the RNeasy Plant Mini Kit (Qiagen, Germany) following the manufacturer’s instructions. RNA concentration and purity were measured using a NanoDrop spectrophotometer, and integrity was verified by agarose gel electrophoresis. Residual genomic DNA was removed using RNase-free DNase I. First-strand cDNA was synthesized from 1 µg of total RNA using the iScript cDNA Synthesis Kit (Bio-Rad, USA) according to the manufacturer’s protocol. qRT-PCR was performed on the QIAquant 96 5plex Real-Time PCR System using Fast SYBR Green Master Mix (Applied Biosystems, USA) in a final reaction volume of 20 µL. Each reaction contained diluted cDNA (1:10), 0.2 µM of each gene-specific primer, master mix, and nuclease-free water. The amplification program was set as follows: initial denaturation at 95 °C for 5 min; 40 cycles of 95 °C for 20 s, 58 °C for 20 s, and 72 °C for 10 s. All reactions were carried out with three biological replicates and three technical replicates. Relative gene expression levels were calculated using the 2−ΔΔCt method with tubulin used as the internal reference gene. Tubulin was chosen because it is a widely used housekeeping gene with consistent expression across tissues and experimental circumstances, including abiotic stress. Previous research in Limonium and other crops under salt stress has shown that tubulin have consistent expression patterns, making them suitable for normalization in gene expression investigations (Wang et al., 2019; Liu et al., 2019; Yang et al., 2025). In addition, Ct values for tubulin showed minimal variation across treatments, supporting its suitability for normalization in the present study. Expression levels in untreated controls were set to 1 for fold-change calculations (Rao et al., 2013). Primer sequences are listed in **Supplementary Table 1**.

### 2.12 Statistical analysis

The statistical analysis was performed using the SPSS software package (version 19.0; IBM, Armonk, New York, USA). The statistical significance was determined using a two-way ANOVA to test the sources of variation and estimate the interaction between the stress treatments and plant varieties. Two-way ANOVA was performed on data derived from three biological replicates (n=3). Significant differences (*P<* 0.05) between the values were determined using Duncan’s multiple range test. Mathematical graphs for all the attributes were drawn utilizing MS-Excel 2019. Correlations between all the variables were quantified with the Pearson correlation coefficient (r) and significance tests were used to calculate the associated p-values with R package. Hierarchical cluster analysis using cluster map function in Anaconda Navigator 2.0.3.

## 3 Results

### 3.1 Salt tolerance threshold determination

**Figure 1:**
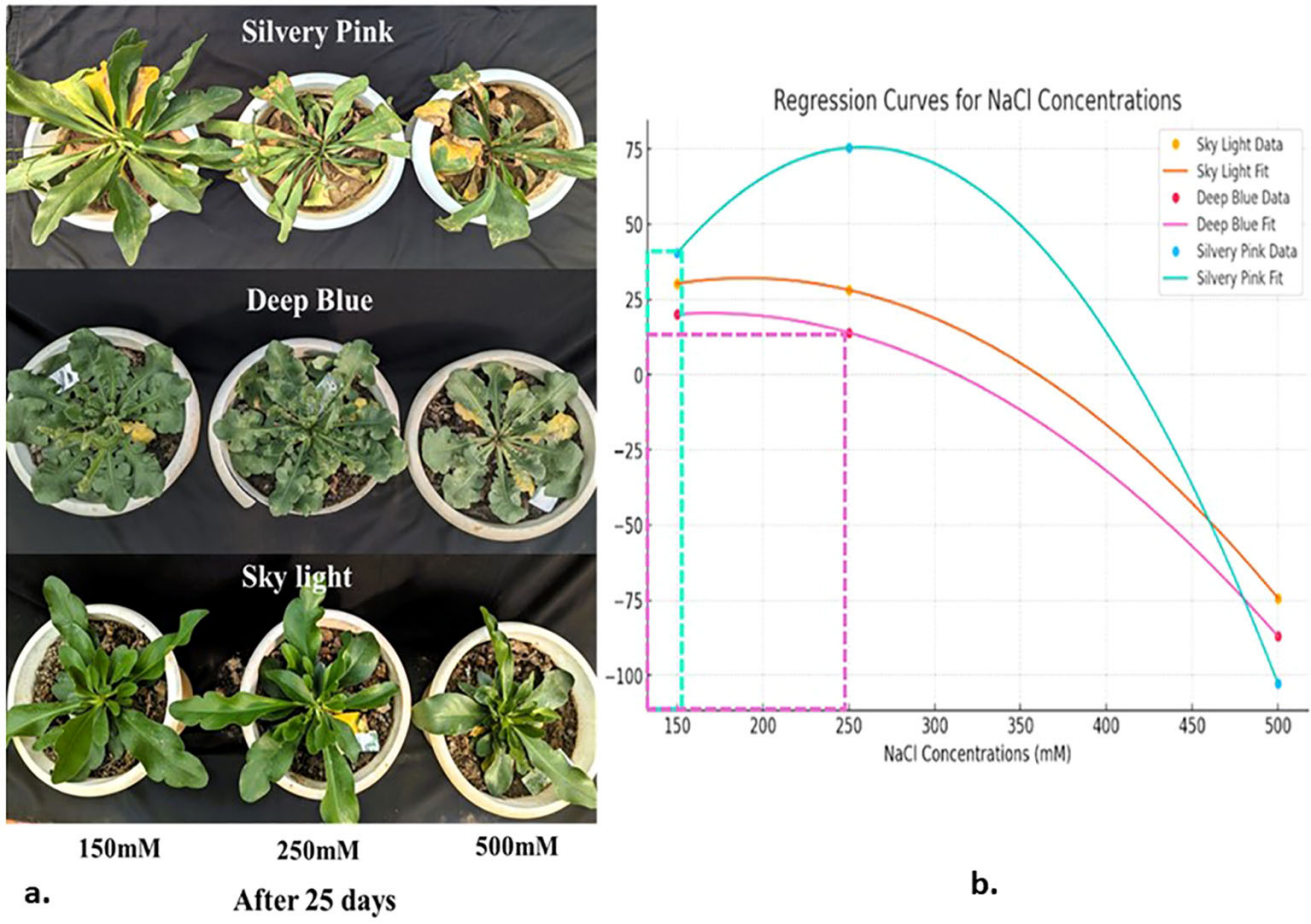
**(A)** Salt-tolerance thresholds of three varieties of limonium species. **(B)** A 50% reduction in biomass compared to the control was used as the standard to determine the salt-tolerance threshold. Changes in dry weight (DW) of the leaves of three varieties under different salt treatments using regression curve.

Plant biomass was used to assess salt tolerance in three *Limonium* varieties: *L. bicolor* (‘sky light’ and ‘silvery pink’) and *L. sinuatum* (‘deep blue’) (**Figure 1A**). Dry weight (DW) was measured under 50, 100, and 250 mM NaCl. Regression analysis showed that ‘sky light’ and ‘deep blue’ retained over 50% of their DW at 250 mM NaCl, indicating higher salt tolerance compared with ‘silvery pink’ (**Figure 1B**). Due to its severe biomass reduction and poor growth under stress, ‘silvery pink’ was excluded from further experiments, as including it could interfere physiological and molecular analyses. Consequently, 250 mM NaCl was selected for subsequent stress treatments, and all further analyses focused on the more resilient varieties, ‘sky light’ and ‘deep blue’, which clearly demonstrated tolerance under the chosen stress level.

### 3.2 Growth and water relations

**Figure 2:**
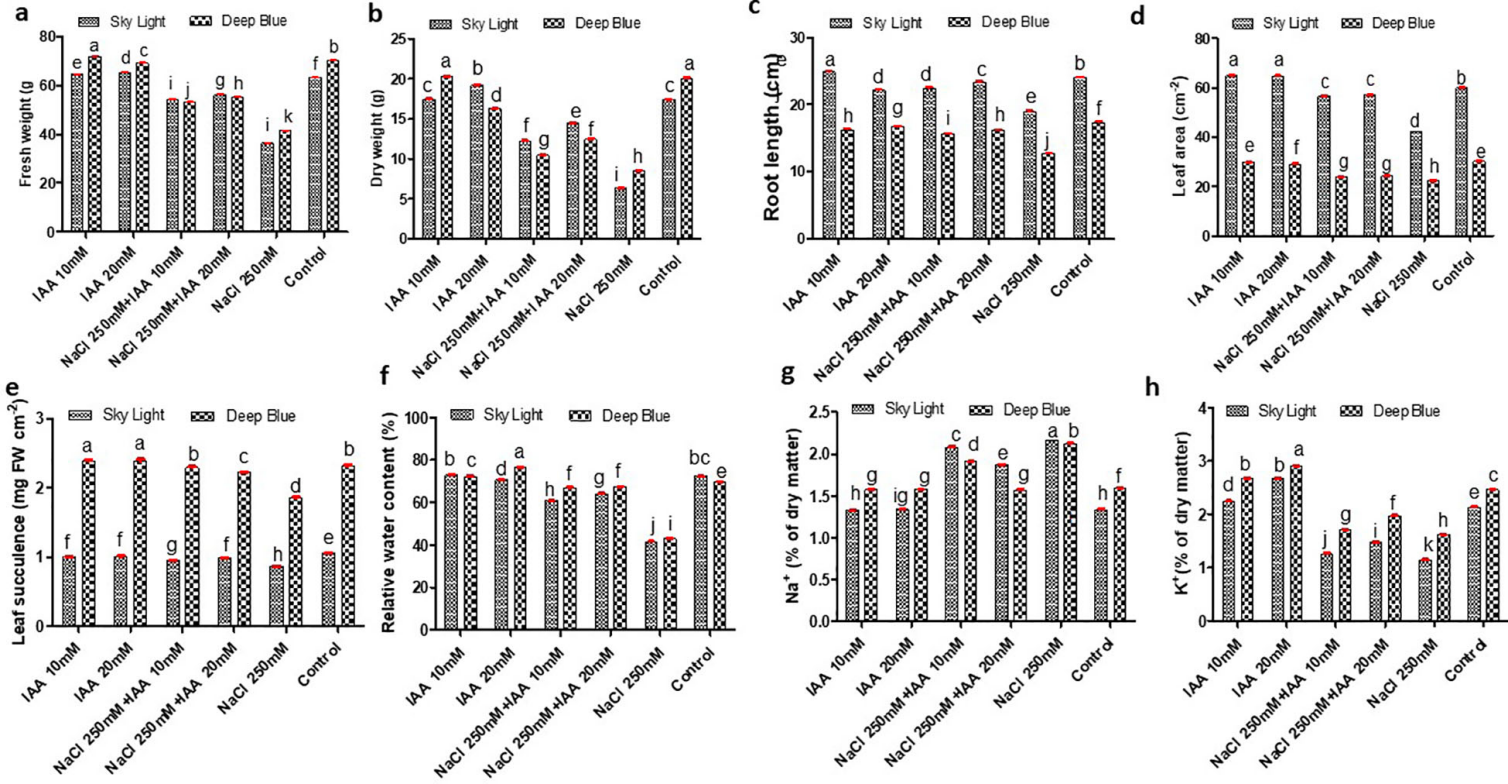
Effect of indole-3-acetic acid concentration (IAA) on **(A)** fresh weight **(B)** dry weight **(C)** root length **(D)** leaf area **(E)** leaf succulence **(F)** relative water content **(G)** Na+ content and **(H)** K^+^ content of sky light and deep blue variety grown under NaCl stress. Different letters indicate significant differences at P < 0.05 according to Duncan’s multiple range test.

Under 250 mM NaCl stress, both ‘sky light’ and ‘deep blue’ exhibited reductions in fresh weight (FW), dry weight (DW), root length, leaf area, leaf succulence and relative water content. In ‘sky light’, IAA priming (20 mM) improved FW by 54.4%, DW by 129.0%, root length by 23.2%, leaf area by 35.5%, leaf succulence by 15.1% and leaf relative water content by 54.19% compared to salt-stressed plants without IAA. Similarly, in ‘deep blue’, FW, DW, root length, leaf area, leaf succulence and leaf relative water content increased by 33.5%, 46.2%, 27.6%, 8.2%, 20% and 56.11% respectively (**Figures 2A–F**).

### 3.3 Mineral element content

Mineral analysis of root tissues showed clear varietal differences in Na^+^ and K^+^ levels under salt stress. A marked increase in Na^+^ content was observed, with ‘sky light’ showing a 63.15% rise compared to control, whereas ‘deep blue’ showed a smaller increase of 33.3%. IAA priming (20 mM) reduced Na^+^ accumulation, with the decrease of 13.82% and 26.41% in ‘sky light’ and ‘deep blue’, respectively. In contrast, K^+^ concentrations declined significantly under NaCl stress; however, IAA application improved K^+^ retention in roots, with increases of 28.9% in ‘sky light’ and 20.85% in ‘deep blue’. These results show variety specific salt responses and suggest that IAA priming helps correct ion imbalances in roots under salt stress (**Figures 2G, H**).

### 3.4 Photosynthetic performance

#### 3.4.1 Gas exchange parameters

**Figure 3:**
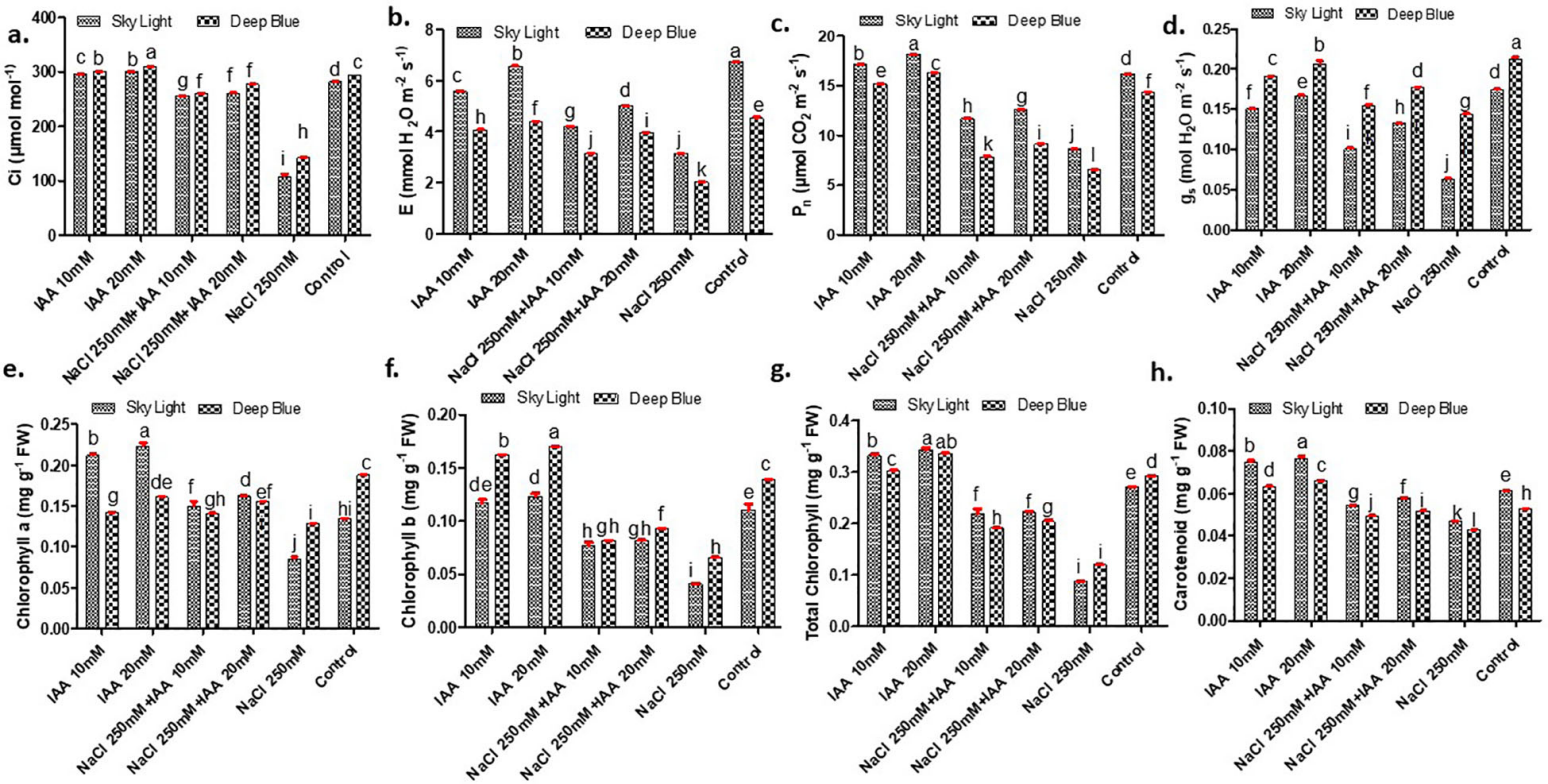
Effect of indole-3-acetic acid concentration (IAA) on **(A)** sub-stomatal CO_2_ concentration (C_i_) **(B)** transpiration rate **(E)**, **(C)** net photosenthetic rate (P_n_) **(D)** Stomatal conductance (gs) (E) chlorophyll “a” (F) chlorophyll “b” (G) total chlorophyll and (H) carotenoid of sky light and deep blue variety grown under NaCl stress. Different letters indicate significant differences at P < 0.05 according to Duncan’s multiple range test.

Salt stress (250 mM NaCl) significantly reduced sub-stomatal CO_2_ concentration (Ci), transpiration rate (E), net photosynthetic rate (Pn), and stomatal conductance (gs) in both varieties compared to the control (**Figure 3**). In sky light, salt stress decreased Ci, E, Pn, and gs by approximately 62%, 53%, 47%, and 64%, respectively. IAA priming mitigated these stress-induced reductions, with a stronger response observed at 20 mM. In sky light, IAA (20 mM) improved all gas-exchange traits under salt stress, restoring Ci, E, Pn, and gs closer to control levels. In deep blue, the corresponding reductions were about 51% for Ci, 56% for E, 55% for Pn, and 32% for gs. A similar trend was observed in deep blue, where IAA priming enhanced these parameters under salinity, although the magnitude of recovery was different among traits. Overall, the results indicate that IAA priming, particularly at 20 mM, effectively alleviated salt-induced inhibition of gas exchange in both varieties (**Figures 3A–D**).

#### 3.4.2 Chlorophyll and carotenoid content

Salt stress (250 mM NaCl) caused a strong reduction in chlorophyll a, chlorophyll b, total chlorophyll, and carotenoid contents in both varieties compared to the control (**Figures 3E–H**). In sky light, salt stress reduced chlorophyll a, chlorophyll b, total chlorophyll, and carotenoids by approximately 37%, 63%, 67%, and 24%, respectively, relative to the control. In deep blue, the corresponding reductions were about 32% for chlorophyll a, 53% for chlorophyll b, 59% for total chlorophyll, and 19% for carotenoids. Priming with IAA (20 mM) alleviated these salt-induced declines in both varieties, leading to clear recovery of all pigment fractions under saline conditions (**Figures 3E–H**).

### 3.5 Oxidative stress and damage

#### 3.5.1 DPPH scavenging activity

**Figure 4:**
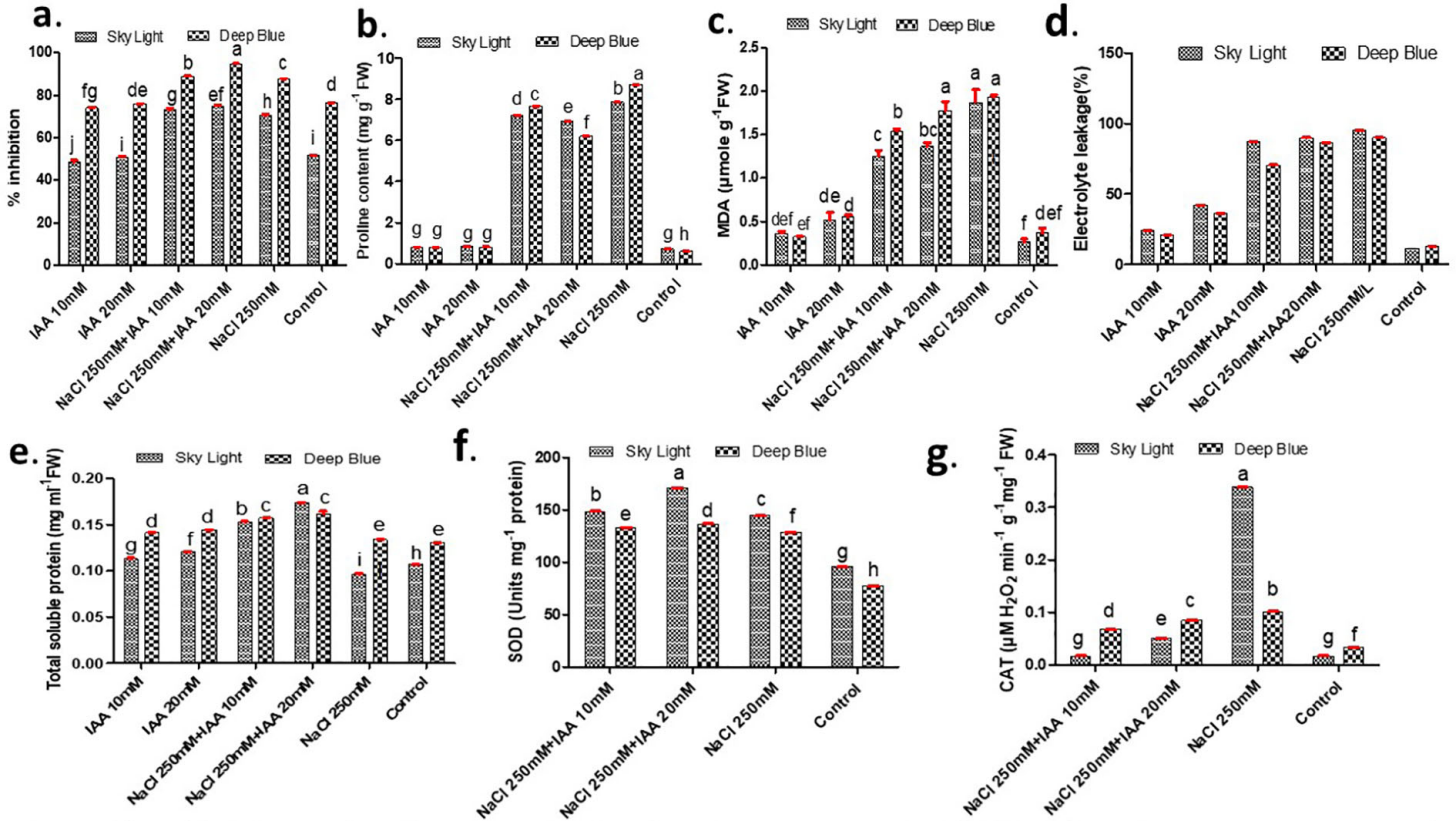
Effect of indole-3-acetic acid concentration (IAA) on **(A)** DPPH assay (% inhibition) **(B)** proline content **(C)** MDA **(D)** electrolyte leakage (%) **(E)** total soluble protein **(F)** superoxide dismutase activity **(G)** catalase activity of sky light and deep blue variety grown under NaCl stress. Different letters indicate significant differences at P < 0.05 according to Duncan’s multiple range test.

Salinity stress (250 mM NaCl) significantly enhanced DPPH radical scavenging activity in both sky light and deep blue, indicating elevated oxidative stress levels. Specifically, DPPH inhibition increased to 57.02% in sky light and 87.66% in deep blue compared to their respective controls. Priming with 20 mM IAA before salinity stress further augmented the antioxidant response, with DPPH inhibition rising to 74.85% in sky light and 94.79% in deep blue compared to salt-stressed plants without IAA (**Figure 4A**).

#### 3.5.2 Proline accumulation

Proline accumulation is well documented adaptive response to osmotic stress. In sky light, proline levels increase from 0.728 mg g^-1^ FW (control) to 7.85 mg g^-1^ FW (salt stress), while deep blue, from 0.608 mg g^-1^ FW (control) to 8.70 mg g^-1^ FW (salt stress). Priming with 20 mM IAA under salt stress reduced proline accumulation, with decreases of 11.71% in sky light and 28.85% in deep blue compared to salt-stressed plants without IAA, respectively (**Figure 4B**).

#### 3.5.3 Lipid peroxidation (MDA levels)

Malondialdehyde (MDA) content, an indicator of lipid peroxidation, increased markedly under salt stress. In sky light, MDA levels significantly increased from 0.26 (control) to 1.86 under 250 mM NaCl. Similarly, in deep blue MDA increases from 0.36 (control) to 1.92 under NaCl stress. 20 mM IAA priming significantly reduced MDA levels, with decreases of 26.88% in sky light and 7.81% in deep blue compared to salt-stressed plants without IAA, respectively (**Figure 4C**).

#### 3.5.4 Electrolyte leakage

Salt stress (250 mM NaCl) significantly increased electrolyte leakage, indicating enhanced membrane permeability in both *Limonium* varieties, with a more pronounced effect observed in sky light compared to deep blue. Priming with 20 Mm IAA under salt stress conditions effectively mitigated membrane damage, reducing electrolyte leakage by 6.28% in sky light and 8.93% in deep blue compared to salt-stressed plants without IAA (**Figure 4D**).

#### 3.5.5 Total soluble protein

Total soluble protein responded differently to salinity stress between the two species. In sky light, total soluble protein content decreased from 0.11 mg ml^-1^ FW (control) to 0.095 mg ml^-1^ FW (salt stress). Priming with 20mM IAA mitigate detrimental effect and improve protein content by 82.1% in sky light and 20.14% in deep compared to salt-stressed plants without IAA (**Figure 4E**).

#### 3.5.6 Antioxidant enzyme activity

Salinity stress induced a significant enhancement in antioxidant enzyme activity in both varieties, with SOD and CAT showing the strongest increases. IAA priming further elevated SOD activity, with increases of 18.09% in ‘sky light’ and 6.09% in ‘deep blue’ compared to salt-stressed plants, indicating enhanced conversion of superoxide radicals into H_2_O_2_. Although CAT activity markedly increased under salinity relative to the control, IAA priming reduced CAT activity in both Limonium varieties. The simultaneous increase in SOD and decline in CAT may suggests that IAA priming lowered oxidative pressure, thereby reducing the requirement for high catalase activity, while maintaining efficient reactive oxygen species detoxification through complementary antioxidant systems. This enzyme pattern points toward improved alleviation of stress and cellular redox balance under salinity (**Figures 4F, G**).

### 3.6 Scanning electron microscopy

**Figure 5:**
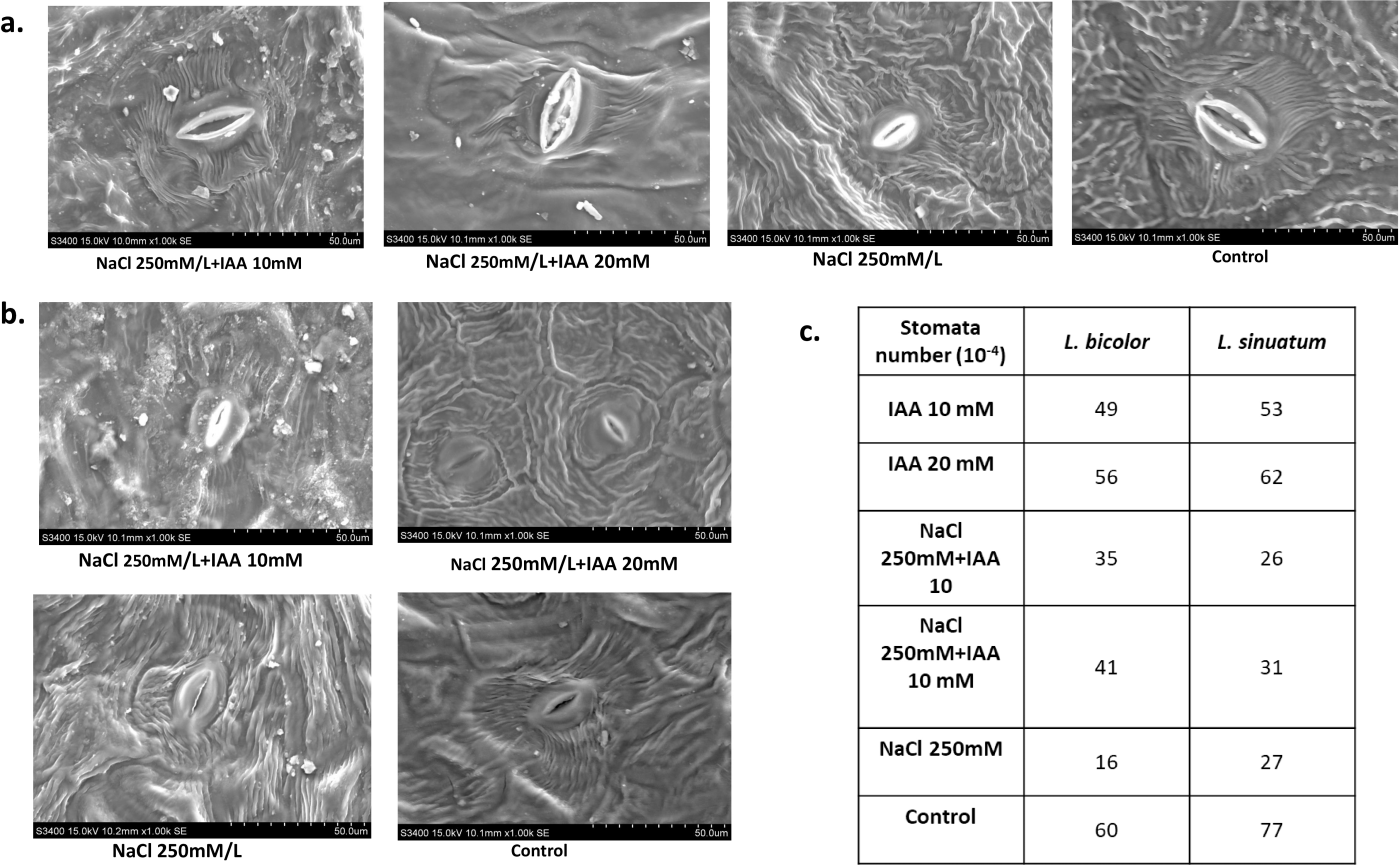
Scanning electron microscope (SEM) images of stomata. Response of stomatal aperture to the application of IAA under salt stress **(A)** Deep blue **(B)** Sky light at 50µm magnification **(C)** table represents the stomatal count of upper surface (adaxial) of both Limonium species.

Both *Limonium* species exhibited a reduction in stomatal density and aperture under 250 mM NaCl stress compared to control plants. However, priming with 20 mM IAA prior to salt exposure significantly enhanced stomatal density and promoted wider stomatal apertures in the leaves of both species, thereby alleviating the closure induced by salinity stress (**Figures 5A–C**).

### 3.7 Cluster-wise analysis of IAA-mediated salt stress amelioration in sky light and deep blue

**Figure 6:**
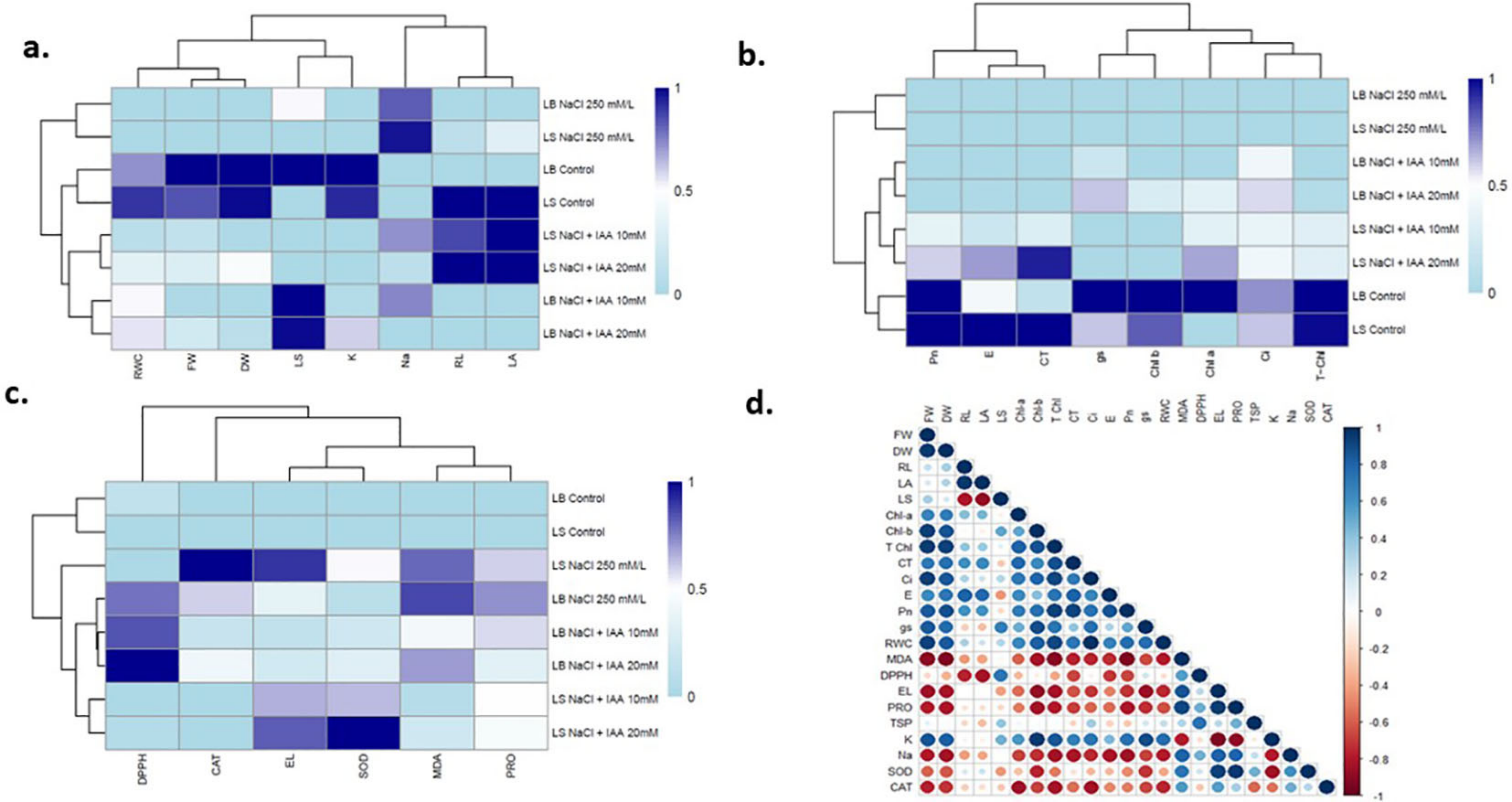
Z score based hierarchical clustering analysis of **(A)** growth and ionic parameters **(B)** photosynthetic performance and **(C)** oxidative stress and antioxidant parameters of two limonium varieties **(D)** Pearson’s correlation analysis between diverse analyzed parameters of limonium species under NaCl stress. Blue and brownish colors indicate positive and negative correlation, correspondingly. FW, fresh weight; DW, dry weight; RL, root length; LA, leaf are; LS, leaf succulence; Chl-a, chlorophyll a; Chl-b, chlorophyll b; T chl, total chlorophyll; CT, carotenoid; Ci, sub-stomatal CO_2_ concentration; E, transpiration rate; Pn, net photosynthetic rate; gs, stomatal conductance; RWC, relative water content; MDA, malondialdehyde; EL, electrolyte leakage; PRO, proline; TSP, total soluble protein; K, K^+^; Na, Na^+^; SOD, superoxide dismutase; CAT, catalase.

The Z score based hierarchical clustering clearly separated the treatments based on salt stress intensity and IAA application in both *Limonium sinuatum* (LS) *and Limonium bicolor* (LB). For growth and ionic parameters (**Figure 6A**), control plants of both species grouped together and showed better growth, higher water status, and greater potassium accumulation, whereas plants exposed to 250 mM NaCl formed a distinct cluster characterized by poor growth and elevated sodium levels. IAA primed salt-stressed plants clustered closer to the controls, showing improvement in root length, leaf area, and water status, with a stronger response at the higher IAA dose (20mM), particularly in LS. Clustering of photosynthetic performance parameters (**Figure 6B**) showed a similar pattern, where control plants were associated with higher chlorophyll and carotenoid contents, improved gas exchange, and higher photosynthetic rates, while salt stress reduced these traits. IAA application under salinity partially restored photosynthetic activity in both species, with greater improvement at the higher dose, especially in LS. For oxidative stress and antioxidant parameters (**Figure 6C**), control plants clustered together with lower electrolyte leakage and lipid peroxidation, indicating minimal cellular damage, whereas salt-stressed plants showed higher MDA, electrolyte leakage, and proline levels along with enhanced antioxidant enzyme activities. IAA treatment helped both species cope better with salt stress by reducing oxidative damage and maintaining a more stable antioxidant response. This protective effect was more pronounced in *Limonium sinuatum* (LS).

### 3.8 Correlation of traits related to salinity tolerance

The correlation heatmap shows clear relationships among growth, physiological, and biochemical traits, with a few exceptions. Growth-related parameters such as fresh weight, dry weight, root length, leaf area, leaf succulence, and chlorophyll fractions show strong positive correlations with gas exchange traits, including Ci, transpiration, photosynthetic rate, stomatal conductance, and relative water content. This indicates that improved photosynthesis and water status directly support plant growth.

In contrast, stress-related indicators such as malondialdehyde, electrolyte leakage, proline and sodium show strong negative correlations with growth (dry weight, fresh weight) and photosynthetic parameters. Their positive association with each other reflects increased oxidative and ionic stress under salinity. Na^+^ content shows a strong negative relationship with growth traits, K^+^ content, photosynthetic pigments (chlorophylls and carotenoids), photosynthetic activity and RWC (**Figure 6D**).

### 3.9 Expression analysis of salt stress related genes through quantitative real-time-PCR

**Figure 7:**
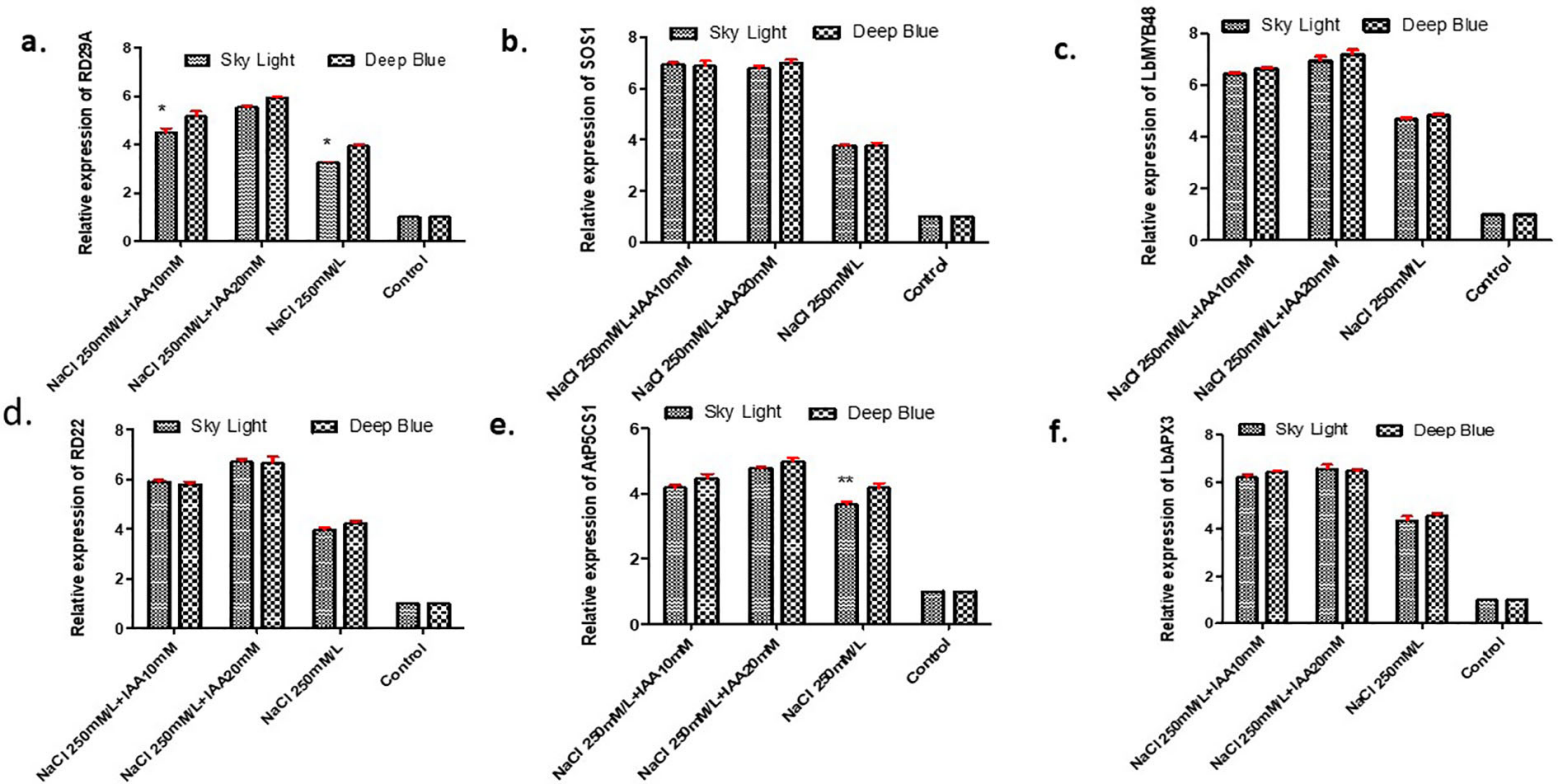
Effect of indole-3-acetic acid concentration (IAA) on the expression of **(A)** RD29A **(B)** SOS 1 **(C)** LbMyB48 **(D)** RD22 **(E)** AtP5CS 1 **(F)** LbAPX3 grown under NaCl stress. The differences were compared by Dunnet’s test with a significance level of *p<.05; **p<.01; versus the control group. The control group (untreated plants) were set to 1 for the 2 ∧ △ △CT calculation.

The expression patterns of key salt stress-responsive marker genes (RD22, RD29A, SOS1, AtP5CS1, LbMYB48, and LbAPX3) (**Supplementary Table 1**) under different treatments were analyzed using qRT-PCR. The results indicated that all targeted genes were upregulated in response to 250 mM NaCl treatment, and a more pronounced induction observed in plants subjected to IAA application along with NaCl. Among the analyzed genes, RD22 exhibited consistently higher expression under salt stress compared to the control (4-fold increase), and 6.7-fold increase in plants treated with 20 mM IAA. Similarly, RD29A, SOS1, AtP5CS1, LbMYB48, and LbAPX3 showed stronger induction under salt stress than in control conditions. The priming of plants with 20mM IAA before exposure of salt stress further amplified the expression of these genes, indicating its positive role in enhancing salt tolerance in *Limonium* (**Figures 7A–F**).

## 4 Discussion

Salinity is a major global stressor that significantly limits plant productivity by disturbing cellular balance. It causes osmotic stress, ion toxicity, and nutrient imbalances, which show plant growth and reduce yields (Nawaz et al., 2010). Ornamental plants, supports a global industry worth $300 billion USD, but they are highly sensitive to salinity as it ruins their aesthetic value through stunted growth and leaf damage (Wang et al., 2025; Azadi et al., 2016). *Limonium* species are widely used in landscaping due to their natural salt tolerance, but this tolerance differs widely among cultivars (Toscano et al., 2023). *Limonium*, is as recretohalophyte and survives in saline soil by using salt glands that actively remove excess ions from leaf tissues. This process prevents toxic ion buildup in photosynthetic cells (Al Hassan et al., 2017; Mi et al., 2021). This feature allows the plant to maintain ion balance and normal metabolism under salt stress and sets *Limonium* different from glycophytes which mainly depend on ion exclusion or vacuolar compartmentalization. *Limonium* can tolerate moderate salinity levels of 100–150 mM NaCl, but growth and physiological processes declined sharply at higher salt concentrations (>200 mM NaCl) (Feng et al., 2015). Salt secretion must therefore work together with osmotic regulation, antioxidant defense, and stress-responsive gene networks to keep cells stable. Identifying the salt tolerance threshold is important to determine the upper limit of tolerance beyond which growth is significantly impaired. In this study, we first quantified the salt tolerance threshold by measuring the dry weight (DW) of three *Limonium* cultivars under increasing NaCl concentrations. The cultivars ‘sky light’ (*L. bicolor*) and ‘deep blue’ (*L. sinuatum*) showed higher tolerance, with a threshold concentration of 250 mM NaCl. This level was defined as the concentration at which plants retained 50% dry weight compared with control. Our findings aligned with Xu et al. (2021), who tested five *Limonium sinuatum* varieties (White, Blue, Pink, Yellow, and Purple) under 250 mmol L^-1^ NaCl and identified the most suitable cultivar for cultivation on saline land.

To mitigate salinity damage, application of plant growth regulators like IAA has become effective approach. IAA is a major auxin that controls cell elongation, vascular development, and stress signaling. Research on crops like wheat, sorghum, moong bean and maize shows that IAA reduces salt stress by improving growth, photosynthesis, and antioxidant defenses (Ali and Ashraf, 2011; Zhang et al., 2024). This study evaluated how IAA priming at 10 mM and 20 mM affects *Limonium* cultivars exposed to 250 mM NaCl stress. In our study, salt induced clear decline in growth and water retentions parameters such as shoot and root growth, leaf area, relative water content and succulence for both cultivars. Reduction of growth due to insufficient water uptake is a common indicator of salt stress reported by Rehman et al. (2022). However, priming with 20 mM IAA significantly countered these effects and helped the plants maintain growth closer to the control level. The results are in agreement with the findings of earlier researchers such as perennial ryegrass and eggplant, where exogenous treatments significantly increased biomass and yields under salt stress (Zhang et al., 2021; Shahzad et al., 2022). IAA application helps maintain turgor pressure and enhances water and mineral uptake, leading to improved root and shoot growth (Díaz-Zorita et al., 2001). This recovery is largely driven by IAA stimulating H^+^ATPases to loosen cell walls for expansion and promotes lateral root development which improves access to water and nutrients (Ma et al., 2022).

In this research, the data suggest that salt stress severely disrupted photosynthetic performance, causing a sharp decline in Chl a, Chl b, and carotenoid pigments at 250 mM NaCl. Sarabi et al. (2017) explained that high levels of Na^+^ and Cl^-^ around the roots can damage photosynthetic pigments due to quick degradation and oxidation of the chlorophyll pigments by ROS, disturbing pigment formation, increasing chlorophyll breakdown, and interfering with pigment-protein structures. However, IAA priming significantly improved photosynthetic performance under these conditions, restoring pigment levels and protecting chloroplast integrity (Rizwan et al., 2018). Similar improvements have been noted in okra (Esan et al., 2017) and mung bean (Rahman et al., 2019a), where IAA enhanced light absorption and water use efficiency. Specifically, by maintaining higher relative water content (RWC) and increasing leaf area, IAA allows plants to capture more sunlight and utilize limited resources more effectively for biomass production (Soundararajan et al., 2017). This mechanism is further supported by Utsumi et al. (2019), who found that acid treatments in cassava improved stress tolerance by regulating stomatal conductance and reducing the flow of toxic ions. Based on these findings, it is reasonable to propose that applying IAA under stress helps maintain photosynthetic efficiency by boosting pigment levels, leaf area, and water content. Furthermore, it enhances water use efficiency by narrowing stomatal openings; this reduces transpiration, which conserves moisture and limits the intake of toxic ions.

Photosynthetic gas exchange parameters (Pn, E, gs, and Ci) in *Limonium* declined markedly under salinity stress due to both stomatal and non-stomatal limitations (Bistgani et al., 2019). The stomata’s opening is closely linked to photosynthesis, which depends on the tissue osmotic potential and cell turgor (Hajiboland et al., 2014). Under salt stress, the initial disruption in water relations typically triggers an increase in ABA levels, leading to guard cell depolarization and stomatal closure to limit transpiration. In this study, the recovery of stomatal conductance following IAA treatment suggests a potential antagonism where IAA overrides ABA-mediated signals. This likely occurs through the suppression of ABA-induced Reactive Oxygen Species (ROS) and the activation of plasma membrane H^+^-ATPase pumps to maintain K^+^ influx and turgor. While these physiological trends align with established IAA-ABA crosstalk models, the lack of direct ABA quantification means this mechanism should be acknowledged as a hypothesis for future work (Qi et al., 2021). Enhanced RWC in IAA-treated plants further supports improved hydration status, which is critical for maintaining photosynthesis and turgor-driven cell expansion. Improved RWC may also reflect increased aquaporin expression and osmotic adjustment, promoting water uptake and retention (Keyvan, 2010).

Salinity-induced oxidative stress, characterized by overproduction of ROS such as superoxide radicals and hydrogen peroxide, disrupts cellular structures, especially lipids, proteins, and DNA. Our results revealed that salt stress significantly elevated MDA and electrolyte leakage, indicating enhanced lipid peroxidation and membrane damage. IAA priming mitigated these effects, as evidenced by reductions in MDA and EL, indicating improved membrane stability. Additionally, IAA application increased the SOD activity and slightly decrease the CAT activity. SOD functions as the primary line of defense in the ROS-scavenging cascade by rapidly converting superoxide radicals (O_2_^-^) into H_2_O_2_, thereby limiting oxidative damage (Zhang et al., 2021; Shahzad et al., 2022). Catalase plays a key role in detoxifying H_2_O_2_; however, the reduced CAT activity observed following IAA priming likely reflects lower cellular H_2_O_2_ accumulation as plants probably ameliorates salt stress due to IAA priming or detoxification may occur via alternative pathways such as the ascorbate–glutathione cycle (APX-mediated). This differential regulation suggests fine-tuning of the antioxidant machinery under IAA priming, prioritizing superoxide detoxification while H_2_O_2_ is efficiently managed through complementary mechanisms, thus indicating stress alleviation and restored redox homeostasis under salinity.

Proline, a multifunctional osmolyte, accumulated under salt stress in both cultivars, indicating an adaptive response that aids osmotic adjustment, limits oxidative stress, and protects cell membrane (Hayat et al., 2012). In comparison to salt stress alone, IAA priming significantly reduced proline buildup. This drop is indicative of IAA’s stress-amelioration response, implying that plants treated with IAA experienced less oxidative and osmotic stress due to improved water status, enhanced antioxidant capacity, and improved ion homeostasis. The lower demand for proline accumulation reflects metabolic balance and better stress tolerance under salinity (Park et al., 2021; Abdel Latef et al., 2021). Furthermore, salt stress caused excessive Na^+^ accumulation and K^+^ loss, disrupting ionic balance and cellular metabolism. IAA priming significantly reduced Na^+^ content and enhanced K^+^ retention, thereby restoring ion homeostasis, likely through modulation of key transporters such as SOS1, NHX1, and HKT1 (Shabala, 2013; Zhang et al., 2021). Similarly, acetic acid treatment increased the K^+^/Na^+^ ratio in ryegrass by upregulating HKT1;1 expression under salinity (Zhang et al., 2021). Scanning electron microscopy further revealed that IAA increased stomatal density and aperture (**Figure 5**) possibly via activation of H^+^-ATPase-mediated K^+^ influx into guard cells, promoting stomatal opening (Mir et al., 2020).

Proteins serve as key biochemical indicators of salinity tolerance. In this study, soluble protein content decreased under 250 mM NaCl stress, in agreement with earlier findings in salt-stressed potato plants (Ejaz et al., 2012). This decline may result from osmotic stress, reduced amino acid availability, enzyme denaturation, or K^+^ depletion, all of which impair protein synthesis under saline conditions (Ayala-Astorga and Alcaraz-Melendez, 2010; Sapre et al., 2022). In contrast, increased protein accumulation under IAA may support nitrogen storage and osmotic adjustment. This response is likely linked to auxin-mediated activation of K^+^ uptake channels, helping maintain ionic balance and enhance tolerance to salt stress (Gull et al., 2023).

Gene expression analysis further supported the physiological and biochemical responses observed under salinity. Salt stress upregulated key stress-responsive genes, including RD22, RD29A, SOS1, AtP5CS1, LbMYB48, and LbAPX3, and this induction was further enhanced by IAA priming, indicating reinforcement of stress defense pathways (Meng et al., 2023; Demirkol, 2023; Rai et al., 2024). RD22 and RD29A function in ABA-mediated stress signaling, while SOS1 encodes a plasma membrane Na^+^/H^+^ antiporter essential for sodium extrusion and ion homeostasis (Ma et al., 2025; Noda et al., 2025). Upregulation of AtP5CS1 supports osmotic adjustment through proline biosynthesis, and increased LbAPX3 expression suggests stronger control of reactive oxygen species (Yang et al., 2024; Han et al., 2022). Importantly, the marked upregulation of LbMYB48 establishes a direct link to the recretohalophytic biology of *Limonium*. LbMYB48 has been shown to promote salt gland development and enhance sodium secretion, thereby improving salinity tolerance (Han et al., 2022). Its stronger induction in IAA-primed plants suggests that auxin treatment may support gland-mediated salt excretion, helping reduce ionic toxicity in leaf tissues. This response indicates that IAA priming supports not only metabolic and antioxidant defenses but also structural adaptations that are central to halophytic survival.

When considered together with the improved K^+^/Na^+^ balance, reduced lipid peroxidation, and enhanced antioxidant activity observed in this study, the transcriptional activation of these genes points to a coordinated stress response operating across molecular, physiological, and structural levels. Such integration highlights a distinctive tolerance strategy in *Limonium*, where hormonal priming may strengthen both internal defense systems and specialized salt-secreting mechanisms.

Taken together, these results demonstrate that IAA priming (20 mM) enhances key physiological, biochemical, and molecular mechanisms associated with salt tolerance in Limonium. Improved growth, pigment retention, photosynthesis, osmotic balance, antioxidant defense, ion homeostasis, and gene regulation collectively underlie the enhanced plant performance under salt stress. Cultivar-specific responses between sky light and deep blue highlight the need for tailored strategies in ornamental crop improvement. The findings indicate that IAA can serve as an effective biostimulant in saline horticulture, improving the resilience and ornamental quality of halophytes under salt-affected soils. This study provides the first comprehensive framework of auxin-mediated salt tolerance in ornamental halophytes and offers both mechanistic insight and practical implications for developing salt-resilient varieties and optimizing floriculture practices under saline conditions.

## 5 Conclusion

This study establishes that salinity stress at 250 mM NaCl substantially impairs growth and physiological performance in *Limonium* species by disrupting ion homeostasis, inducing oxidative damage, and compromising membrane stability. Application of IAA, particularly at 20 mM, significantly alleviated these adverse effects by enhancing osmotic balance, preserving photosynthetic efficiency and pigment stability, reducing oxidative damage, and modulating the expression of key stress-responsive genes. IAA priming improved ion selectivity, increased antioxidant enzyme activities, and conferred greater membrane integrity, underscoring its potential role as a regulatory molecule in salt stress adaptation. These findings underscore the functional relevance of IAA-mediated priming in ornamental species and provide mechanistic insight into its ameliorative effects under high salinity. Further exploration of IAA’s molecular targets and its crosstalk with other hormonal pathways is required to better elucidate the integrative signaling networks governing stress resilience in halophytes such as limonium. Also integrating transcriptomic and metabolomic analyses could further elucidate IAA’s role in stress adaptation and its potential application in sustainable saline horticulture.

## Data Availability

The original contributions presented in the study are included in the article/**Supplementary Material**. Further inquiries can be directed to the corresponding author.
