# Supplementary material for: Hormonal priming with indole-3-acetic acid modulates antioxidant defense, gene expression, and physiological responses under salinity in Limonium spp
Source: Front Plant Sci. 2026 Apr 10;17:1765513. doi: 10.3389/fpls.2026.1765513 (PMC13105954; doi:10.3389/fpls.2026.1765513)
Supplement: Supplementary file 1 [file DataSheet1.pdf]

Supplementary Table 1: Primer Sequences used for RT-qPCR

Name of Primers	Forward primers	Reverse primers
RD29A	5-ATCACTTGGCTCCACTGTTGTTC-3	5-ACAAAACACACATAAACATCCAAAGT-3
RD22	5-ATAATCTTTTGACTTTCGATTTTACCG-3	5-CTTGGACGTTGGTACTTTTCTCG-3
AtP5CS1	5-TAGCACCCGAAGAGCCCCAT-3	5-TTTCAGTTCCAACGCCAGTAGA-3
SOS1	5-TTCATCATCCTCACAATGGCTCTAA-3	5-CCCTCATCAAGCATCTCCCAGTA-3
LbTUBULIN	5-GGTTGAGTGAGCAGTTCAC-3	5-GATAACCAGCCACACCTTAGC-3
LbMYB48	5-GCTCAAGAAAGGAAACAA-3	5-ACGAGATGGAAGAAGAAC-3
LbAPX3	5-CCGTGAAGATGAAGTATC-3	5-GGACAAAGTCTATGGTTG-3
